# Private well water stewardship in rural Georgia

**DOI:** 10.1371/journal.pone.0307281

**Published:** 2024-09-19

**Authors:** J. Edward Dotherow, Bettye Apenteng, Andrew Hansen, Asli Aslan

**Affiliations:** 1 Department of Public Health, the University of Texas at Tyler Health Science Center, Tyler, Texas, United States of America; 2 Institute for Water and Health, Georgia Southern University, Savannah, Georgia, United States of America; 3 Department of Health Policy and Community Health, Georgia Southern University, Statesboro, Georgia, United States of America; 4 Department of Biostatistics, Epidemiology and Environmental Health Sciences, Georgia Southern University, Statesboro, Georgia, United States of America; United States Environmental Protection Agency, UNITED STATES OF AMERICA

## Abstract

This study sought to identify the psychosocial influences on the practice of well stewardship behaviors (water testing, water treatment, and well maintenance) in rural Georgia, USA. Three interventions (education, the provision of household water treatment systems [HWTS], and both education and HWTS) were evaluated using a four-group, randomized controlled trial. A total of 64 private well owners completed a pretest measuring psychosocial factors and stewardship behaviors before receiving an intervention. Following a 104-day waiting period, participants completed a posttest and interviews were conducted to identify the barriers and facilitators to use (S1 File). Pretest results showed that 34% of well owners have ever tested their water and that only 25% treat their water before consumption. The education-only intervention showed no influence on stewardship behaviors, resulted in no new water tests and had no impact on psychosocial factors. The HWTS-only intervention had no significant effect on testing and treatment behaviors, though it had a significant effect on abilities (R^2^ = .87, p< 0.05) and self-regulation (R^2^ = 1.0, p<0.01). The intervention of both education and HWTS had no effect on testing and no significant effect on treatment behaviors, though had a significant effect on abilities (R^2^ = .84, p<0.05) and self-regulation (R^2^ = .93, p<0.05). This study identified three barriers to the use of HWTS: beliefs, knowledge, and functionality. Two factors (piece of mind and ease of use) were identified as facilitators to the use of HWTS. The results of this study indicate that providing water treatment systems does not guarantee use and that current educational efforts provided by state and local health departments may be ineffective.

## Introduction

In the United States, an estimated 13.7 million households rely on private wells as their primary source of drinking water, of which 8.7 million are outside of micropolitan areas [1, 2]. Private wells are susceptible to chemical and microbial contamination from natural and human sources including stormwater run-off, agricultural and industrial activities, septic system leeching, and geogenic processes [3–5]. Long-term exposure to waterborne hazards can result in serious health conditions, including gastrointestinal diseases, endocrine disruption, reproductive problems, birth defects, preterm birth, and bladder, colon, kidney, and thyroid cancers [6–10].

Because many contaminants originate from the same source, these hazards are usually detected together. For example, a study of over 2000 private wells that had nitrate concentrations higher than 1 mg/L also had elevated levels of pesticides and volatile organic compounds [11]. Waterborne pathogens in household plumbing systems also pose health risks, specifically to vulnerable populations [12, 13]. For example, *Legionella* and Pseudomonas species are resilient and known to survive and even thrive in low or no chlorine environments [13–16].

To protect citizens from exposure to contaminated water, public water systems (those serving at least 25 people a year for 60 days or having at least 15 service connections) are regulated under the Safe Drinking Water Act (SDWA). However, the protections offered by the SDWA do not extend to private well owners [17]. Private well owners are responsible for monitoring and ensuring their water quality. While neither the USEPA nor states regulate private wells, they recommend that owners conduct annual water testing for bacteria, nitrates, total dissolved solids, pH, and other substances known to be common in the area’s groundwater [18]. More frequent testing at wells providing water to the elderly, children, or other vulnerable populations. If a water quality analysis reveals one or more contaminants exceeding federal standards, the USEPA recommends treating the water to prevent exposure. Finally, it is recommended that well owners regularly service and maintain their wells to help prevent contamination through breaks and intrusion, as well as ensure the delivery of water [18].

Research on how these recommendations are pursued by private well owners is limited, with most behavioral studies in high-income countries originating in Canada or the Northeast area of the United States, [19–21]. Further, the majority of these studies have been in areas with known arsenic levels, where there is already some level of community knowledge on the health impacts of poor water quality and therefore higher environmental literacy. To determine the psychosocial factors (individual and societal characteristics) influencing stewardship behaviors, the RANAS model (Risks, Attitudes, Norms, Abilities, Self-Regulation) was used [22]. The RANAS model incorporates personal perceptions into the normative, social, and environmental contexts. This model is appropriate as it identifies the psychosocial factors necessary for a behavior to be conducted. The RANAS model was developed specifically to address water, sanitation, and hygiene issues and has been used developed and developing countries alike [22]. This model has been applied in previous studies investigating stewardship behaviors. In central Maine, a RANAS-based survey was conducted to understand water testing and treatment behaviors. The study found that knowledge of the risks associated with arsenic exposure, the ability to find someone to test their water, and the testing behaviors of neighbors were predictors of water testing, while low treatment rates were associated norms, attitudes, and abilities [21].

The purpose of this study was to identify the stewardship behaviors (water testing, water treatment, well maintenance) practiced by private well owners in a rural agricultural communities located in the Southeastern United States, specifically South Georgia. We identified the primary psychosocial factors that drive those behaviors and the barriers and facilitators to the use of household water treatment systems. Furthermore, we evaluated the efficacy of three interventions: education using existing state and local health promotion materials, the provision of household water treatment systems, and the combination of education and the provision of household water treatment systems in increasing stewardship behaviors. Finally, we sought to identify the barriers and facilitators to the use of POU treatment systems.

## Materials and methods

### Study area

**Fig 1:**
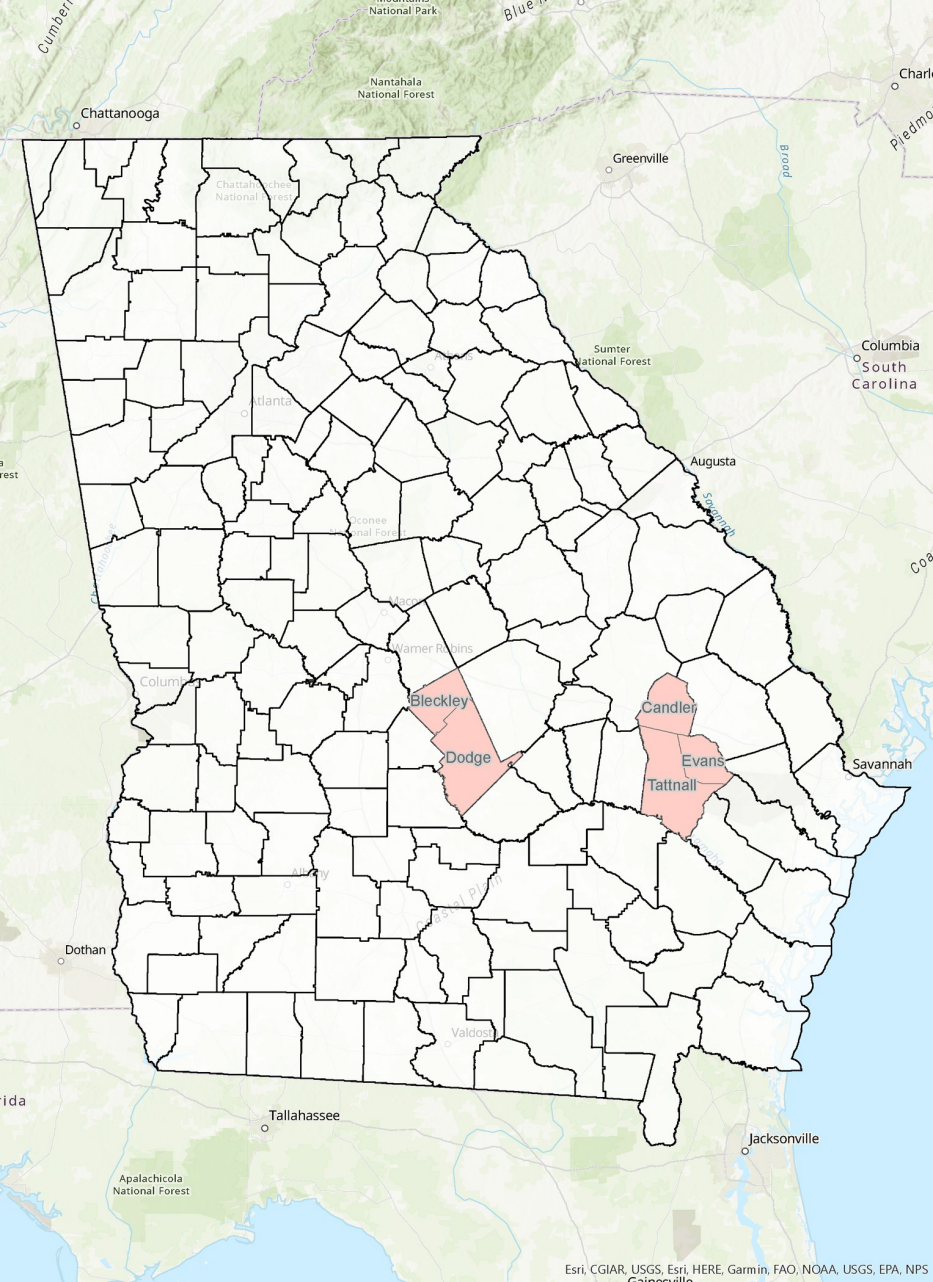
Study locations. (The map was created with open-source data brought into the ArcGIS Online mapping software. Overlays were colored to highlight targeted counties. The output graphic was created utilizing the native .pdf print function in ArcGIS Online).

Southeast Georgia was selected as the study area (Fig 1). A total of 64 residents from the rural agricultural counties of Evans (n = 35), Dodge (n = 25), Candler (n = 1), Tattnall (n = 1), and Bleckley (n = 2) participated in this study. Evans and Dodge Counties are rural, agriculture-centric areas in Georgia, USA, with populations of 10,638 and 20,452, respectively [23]. Both Evans and Dodge counties are primarily white (55.8% and 64.3%) and have median household incomes well below the state median ($72,700) of $51,000 and $42,700, respectively [23].

Candler, Tattnall, and Bleckley counties are rural, agricultural-based communities with populations of 10,985, 25,365, and 12,955, respectively. Median household incomes are similar between Candler ($42,600) and Tattnall ($45,200) counties, with Bleckley County’s being moderately higher ($51,400), though still below the state median of $72,700. Candler (60.6%), Tattnall (57.2%), and Bleckley (67.3%) counties are predominantly white communities [23].

### Recruitment

Recruitment began on December 10, 2020, and ended on March 1, 2021. Eligible participants must have been private well owners in rural GA and relied on that well as their primary source of drinking water. A mix of random and convenience sampling was employed. A map of Evans County, GA, was divided into square miles grids, and each grid was given an identifier (A1, A2, etc.) and entered into an Excel spreadsheet. Grids and areas of the county containing municipal water supplies were excluded. Municipal water supply areas were determined based on the presence of a centralized, government operated water system, based on the state public water system permit list (https://gadrinkingwater.net/DWWPUB/). Grids were then identified using the random selection feature in Excel. Once the grids were selected, addresses and occupant names for potential participants were identified using Google Earth and qPublic.net, resulting in 294 potential participants. White Pages (https://www.whitepages.com/) was then used to identify telephone numbers for those potential participants.

Of the 294 potential participants identified, telephone numbers were found for 174, who were then contacted by phone, given a description of the study, and invited to participate. Informed consent was collected verbally after participants were informed of all potential risks and documented on the participant information spreadsheet. All participants were mailed a copy of the informed consent form. The study was approved by the Georgia Southern University Institutional Review Board affirming it was conducted in compliance with all relevant ethical standards.

Cancer Association Relief Effort and Support (CARES) Foundation members were asked to reach out to potential participants to assist in recruitment. The CARES Foundation is a local group of people that funds and promotes research aimed at finding a cure or reducing the risks of cancers. CARES members were given a list of potential participants and a short description of the study, who then called potential participants and relayed the information. The foundation then emailed the research team a list of those that either agreed to participate or were interested in learning more about the study. Lacking sufficient participation during the COVID-19 restrictions, the study area was expanded to Dodge County, GA. A flyer describing the study was distributed to churches and community centers. Additionally, the flyer was posted on the social accounts of community centers and community leaders. The few participants from Bleckley, Tattnall and Candler counties received recruitment materials from Evans and Dodge counties and volunteered to participate in the study.

### Methodology

This study used a sequential mixed-methods approach and a four-group randomized control trial with a pre and posttest to evaluate the effectiveness of the interventions on psychosocial factors and health behaviors against a comparison group. At the beginning of the study every participant completed a pretest. The pretest measured individual and social factors known to influence well stewardship behaviors based on the RANAS Model (risks, attitudes, norms, abilities, and self-regulation) as well as the current stewardship behaviors currently being practiced. RANAS-based questions were measured using a 5-point Likert scale ranging from 1 (strongly disagree) to 5 (strongly agree).

Risk was measured by assessing the knowledge, perceived susceptibility, and perceived severity of water-related environmental health issues of participants. Attitudes was measured with questions assessing the emotions (sensory perceptions like taste and smell) and beliefs regarding the quality of well water. Injunctive, descriptive, and personal normative behaviors were measured for the Norms construct. Abilities were assessed with questions measuring the action knowledge and self-efficacy or participants. Finally, Self-regulation was measured with assessing the ability to remember and create an action plan to test and treat water, as well as their commitment to water testing and treatment.

Participants were randomly assigned to one of 4 groups. Participants in group 1 received educational materials, group 2 received a point-of-use water treatment (POU) system (a faucet-mounted activated carbon filter), group 3 received both educational materials and point-of-use water treatment system, and group 4 served as the control group.

After mailing the interventions to participants, the research team waited 104 days before contacting the participants to take the posttest. The 104-day waiting period was determined as the filters for POU devices used in this study generally need to be replaced after 90 days. By waiting an additional 2 weeks, participants were forced to decide whether they would continue using the POU devices. After the waiting period, participants completed the posttest. The posttest was identical to the pretest with the addition of open-ended questions to inquire about the barriers and facilitators to the use of the POU devices and opinions on the educational materials.

### Interventions

#### Education

The educational intervention consisted of brochures and flyers developed by the USEPA and Georgia Department of Public Health (GDPH). The USEPA promoted annual water testing and water treatment if necessary. The GDPH materials promoted annual water testing, water treatment for contaminants of concern, and the benefits of well maintenance. Both materials were available in local public health departments in paper format (S2 and S3 Files). The materials were downloaded from the agency websites. The content of the materials was not modified, however, the USEPA flyer was reformatted for size.

#### Point-of-use household water treatment systems

Activated carbon faucet-mounted filters were purchased and given to participants. The systems were NSF certified (standards 42/53/401) to remove chemicals including lead and pesticides (Atrazine, Simazine, etc.), and included the system and one filter, with replacement filters available for purchase at local stores and online.

### Data analysis

#### Quantitative analysis

The data collected in this study were analyzed using Stata v.15 (https://www.stata.com/stata15/). The demographic profile for each group was assessed using descriptive statistics. A chi-square analysis was conducted to assess the effectiveness of the randomization process in balancing the groups on baseline demographic characteristics and psychosocial factors, as measured using the RANAS Model.

Respondents stated their level of agreement with each RANAS statement on a scale of 1 (strongly disagree) to 5 (strongly agree). The set of questions collectively assessing the RANAS constructs had acceptable reliability in the pre-and post-tests (Cronbach alpha: pre-test = 0.7964 and post-test = 0.8953). For each RANAS construct, a composite score was created as the mean across all construct-related items or questions. Mean construct scores from pre- and post-intervention were subsequently calculated as a whole and by group assignment. Mean scores were interpreted as follows: very low (1–2), low-neutral (2.1–3), moderate (3.1–4), high (4.1–5). Significant differences in mean variable scores between groups for each RANAS construct were identified using an ANOVA analysis.

The association between psychosocial (RANAS variables) factors and the binary outcomes of water testing and water treatment were examined by analyzing the pre-intervention data using a chi-square test. RANAS variables associated with testing and treatment at a p<0.25 were then included as control variables in a multivariable logistic regression model to determine the independent psychosocial drivers of water treatment and treatment, respectively.

*Intervention effect*. Following the posttest, the effects of the interventions on RANAS variables were identified using linear regression and a 2-by-2 factorial design. Separate regression models were conducted with each RANAS construct as an outcome, measured with a mean composite score. Each model included the time variable (post vs. pre), the intervention group, and the interaction between the intervention group and time variables. The intervention effect was determined using the coefficients from the interaction terms.

The effect of the intervention on the binary outcome of water treatment was also examined using multivariable logistic regression. Using a 2-by-2 factorial design, the model included the time variable (post vs. pre), the intervention group, and the interaction between the intervention group and time variables. The intervention effect was determined by evaluating the coefficients from the interaction term. Additionally, the model controlled for psychosocial predictors found to be independently associated with water treatment in the analysis of the pre-intervention data afore described. The study was unable to assess the impact of the intervention on water testing due to the limited number of testing behaviors occurring in the post-intervention phase.

The multiple imputation method was used to account for missing data (n = 8). Multiple imputation was accomplished using Stata’s (version 15) mi package and a multivariate normal distribution (mvn) model. The mvn model uses Markov Chain Monte Carlo (MCMC) procedures on the assumption that all variables in the imputation model have a joint multivariate normal distribution. The MVN model has been shown to produce reliable estimates even under conditions of normality assumption violations [24].

Additionally, regression analyses were conducted using completed cases data sets (pre and post-test) and available cases data sets (all available data) as sensitivity analyses. All analyses were clustered at the individual level to account for the repeated measurement. Statistical significance was assessed at a p<0.05 level, using two-tailed tests.

#### Qualitative analysis

The qualitative data collected in this study were recorded and transcribed using Temi (https://www.temi.com). The data was then cleaned, analyzed, and coded inductively. Codes were then collated into themes and subthemes. Once identified, themes and subthemes were reviewed for code coherence. A final review of all data was then done to identify any potentially missing codes, themes, and subthemes.

## Results and discussion

Sixty-four residents from the rural agricultural counties of (Evans (n = 35), Dodge (n = 25), Candler (n = 1), Tattnall (n = 1), and Beckley (n = 2)) participated in this study. Participants’ demographic characteristics were: White (91%), male (59%), and over half (56%) had completed a technical school degree or university degree. Most participants (68%) were aged 56 years or older. Most participants were employed full-time (66%) and had an annual income of more than $100,000. Table 1A presents the demographic distribution of participants in each group. Following random assignment, participants did not differ by group on demographic characteristics (Table 1B).

**Table 1:** **a:** Demographic Data. **b**: Participant Characteristics by Intervention Group.

a					
Race			White 91%		
			Black 6%		
			Hispanic 3%		
Gender			Male 59%		
			Female 41%		
Age			25–35 6%		
			36–45 8%		
			46–55 17%		
			56–65 24%		
			65 and older 44%		
Education			Some High School 1%		
			High School Diploma/ GED 27%		
			Some College 16%		
			Technical or Community College 9%		
			Bachelor’s Degree 30%		
			Graduate Degree 17%		
Employment			Full-Time 66%		
			Part-Time 5%		
			Retired 26%		
			Unemployed 3%		
Income			$20K- $35K 4%		
			$36K- $51K 8%		
			$52K- $67K 15%		
			$68K- $83K 10%		
			$84K- 100K 10%		
			More than $100K 37%		
**b**					
Demographic	Group 1 n (%)	Group 2 n (%)	Group 3 n (%)	Group 4 n(%)	p-Value
White	14 (88%)	15 (88%)	14 (88%)	15 (100%)	0.565
Age < 55 years	7 (44%)	5 (31%)	3 (19%)	5 (33%)	0.507
Tech degree or higher	8 (50%)	11 (65%)	9 (56%)	8 (53%)	0.850
> $65k year	2 (18%)	6 (40%)	7 (47%)	4 (36%)	0.505
Full/ Part-time Employment	13 (81%)	13 (77%)	10 (63%)	9 (60%)	0.483

Group1: Education intervention

Group 2: Filtration intervention

Group 3: Education & filtration intervention

Group 4: Control group

### Practice of stewardship behaviors

Pre-intervention data showed that of the 64 participants, only 34% (n = 22) had tested their water prior to this study. For those who tested their water, it was found to be infrequent. Most had only tested their water once (n = 18), while only three tested every 5–10 years and one participant had tested in the last 10 years). Research has shown that overall water testing rates vary across regions and populations of well owners. Studies have shown that a majority of well owners have tested their water at least once, but less than 35% conduct annual testing as recommended by the USEPA [25–29]. Irregular testing behaviors like those found in this study are more common, with well owners generally testing their water every 5–10 years [21, 25, 26, 29, 30]. The irregular testing patterns practiced by participants could also be explained by variations in outreach and engagement by local authorities. Wait et al. (2020) found that local health departments (LHDs) are not uniform in information sharing or resource distribution. The study found that of 64 LHDs surveyed, roughly half (52%) maintain website information and 30% distributed resources at local events [31].

Likewise, water treatment was practiced by few well owners. Only 25% (n = 16) treated their water before drinking, with the most common type of treatment being a refrigerator filter (n = 14). Three participants used point-of-entry sediment filtration systems, and three participants reported only drinking bottled water. The treatment rates found in this study were lower than the 34%-64% rates found in other studies [27, 30, 32], though some have found treatment rates below 10% [21, 25, 26, 29, 30]. Only three studies found treatment rates above 50% among the study population [20, 29, 30].

Like testing and treatment rates, few participants performed regular maintenance on their wells. Only 16% (n = 10) stated they conducted regular maintenance activities. The most common activities performed were part replacement/ updating (n = 10) and pipe flushing (n = 9).

### Psychosocial factors and stewardship behaviors

#### Pre-intervention

Pre-intervention test scores revealed that overall, participants were moderately knowledgeable and aware of the risks surrounding well water (M = 3.74, SD = 0.72) (Table 2). Participants agreed that the quality of water can change over time (M = 4.27, SD = 0.92) and water can contain dangerous chemical and microbial contaminants (M = 4.25, SD = 0.97). This finding differs from those of other studies, where risk perceptions, knowledge of potential contaminants and associated health risks of exposure were low [33, 34]. However, participants were more moderate in assessing the risks of water contamination (M = 3.23, SD = 1.41) and susceptibility to exposure to contaminated water (M = 3.35, SD = 1.47).

**Table 2:** Preintervention RANAS model mean scores (SD).

Construct	Construct Element	Mean (SD)
Risk		**3.74 (0.72)**
	Knowledge	3.92 (1.22)
	Perceived Susceptibility	3.35 (1.47)
	Perceived Severity	3.94 (1.23)
Attitudes		**3.75 (0.56)**
	Beliefs	3.46 (0.94)
	Emotions	4.20 (0.98)
Norms		**2.43 (1.01)**
	Descriptive	2.25 (0.91)
	Injunctive	1.83 (0.97)
	Personal	4.75 (0.81)
Abilities		**3.08 (0.65)**
	Action Knowledge	2.78 (1.65)
	Self-efficacy	3.52 (1.47)
Self-regulation		**2.62 (0.38)**
	Action Planning	2.66 (1.52)
	Remembering	2.73 (1.45)
	Commitment	2.48 (1.26)

Similarly, participants were generally moderate (M = 3.75, SD = 0.56) in their attitudes towards and beliefs regarding the quality of well water (see Table 2). Participants generally believed in the high quality of well water (M = 3.80, SD = 0.92). Participants strongly agreed that well water tastes better than municipal water (M = 4.64, SD = 0.69) and to a lesser degree was less odorous (M = 3.75, SD = 1.03). Participants were more moderate when reflecting on the affordability of water testing (M = 3.44, SD 0.90).

Similar to other studies, our pre-intervention data showed a weak culture of well stewardship [20, 21, 33]. Participants did not believe that their neighbors (M = 2.28, SD = 1.01), families (M = 2.23, SD = 0.82), or friends (M = 2.27, SD = 0.85) tested their water. Likewise, participants did not believe that their friends (M = 2.33, SD = 1.09), families (M = 2.20, SD = 0.83), or neighbors (M = 2.19, SD = 0.83) treated their water, nor did they feel as though they were expected to test or treat their water (M = 1.83, SD = 0.97) (Table 2).

Abilities related to water testing and treatment (self-efficiency, action knowledge) were neutral, as little agreement between participants was found. Among all participants, the overall abilities mean score was 3.08 (SD = 0.65). Participants were moderately confident in their ability to find someone to test their water (M = 3.75, SD = 1.33), though they were unsure what to test for (M = 2.23, SD = 1.39) or how to treat their water (M = 2.56, SD = 1.64). The nuanced nature of stewardship abilities is well documented, as well owners know how to test and treat their water in general while lacking critical operational knowledge [26, 30].

Interestingly, the mean action knowledge (knowing to how to treat water) score for those who treat their water was 3.25, indicating a neutral take on their knowledge of how to treat water. Most participants using refrigerator filters initially responded “no” when asked if they treat their water. Only when treatment technologies were listed did they respond in the affirmative. Knowledge and attitudes influence each other and can be a barrier to the use of treatment systems. In North Carolina, well owners’ misperceptions regarding water treatment systems stopped them treating their water despite feeling vulnerable to contaminated water [35].

Prior to the intervention participants had no plans for testing their water (M = 2.84, SD = 1.47), nor did they plan on treating their water before consumption (M-2.48, SD = 1.54). Commitment to testing was low (M = 2.66, SD = 1.38), as was a commitment to treatment (M = 2.30, SD = 1.09) (Table 2).

The analysis of pre-intervention data indicated that attitudes and abilities were the primary drivers for water testing (Table 3). Abilities (self-efficacy and action knowledge) were found to be associated with testing (OR = 2.103, 95% CI [0.863, 3.196], p = 0.027). The direction of association between attitudes (affordability of testing and sensory perceptions) and water testing was positive. However, this was not statistically significant at the p<0.05 level (adjusted odds ratio [OR] = 5.708; 95% CI [0.873, 37.318], p = 0.0690). Risks, norms, and self-regulation were also not found to be associated with water testing behaviors.

**Table 3:** Psychosocial factors associated with water testing (pre-intervention).

	Unadjusted			Adjusted		
Variable	OR	p-value	95% CI	OR	p-value	95% CI
Risk	0.630	0.488	0.171–2.320	0.855	0.830	0.199–3.676
Attitudes	4.032	0.085	0.824–19.728	5.708	0.069	0.873–37.318
Norms	1.480	0.364	0.634–3.454	1.150	0.800	0.388–3.406
Abilities	2.245	0.006	1.256–4.016	**2.103**	**0.027**	1.088–4.065
Self-Regulation	1.841	0.039	1.029–3.291	1.661	0.128	0.863–3.196

R = odds ratio, CI- confidence interval

Models adjusted for clustering at the individual level.

The analysis found that of all five constructs, only self-regulation was associated with water treatment (Table 4). Self-regulation (action planning and commitment) was associated with water treatment (OR = 2.064, 95% CI [1.013–4.204], p = 0.046). The positive relationship between norms (the perception of other’s actions and expectations) and water treatment was not statistically significant at the p<0.05 level (OR = 3.008, 95% CI [0.908, 9.968], p = 0.071). Furthermore, the study did not find an association between water treatment and risks, attitudes, or abilities.

**Table 4:** Psychosocial factors associated with water treatment (pre-intervention).

	Unadjusted			Adjusted		
Variable	OR	p-value	95% CI	OR	p-value	95% CI
Risk	0.518	0.359	0.127–2.112	0.603	0.519	0.129–2.801
Attitudes	0.580	0.497	0.120–2.795	0.834	0.842	0.140–4.950
Norms	2.679	0.051	0.995–7.211	3.008	0.071	0.908–9.968
Abilities	1.189	0.526	0.696–2.030	0.784	0.477	0.403–1.528
Self-Regulation	2.034	0.032	1.061–3.898	**2.064**	**0.046**	1.013–4.204

OR = odds ratio, CI- confidence interval

Models adjusted for clustering at the individual level.

### Interventions and effects

Of the 64 study participants, 16 participants were assigned to group 1 (education intervention), of which 14 completed the posttest, two of which were lost to follow-up. Seventeen participants were assigned to group 2 (the water filter intervention), of which 13 participants completed the posttest, and four participants were lost to follow-up. Sixteen participants were assigned to group 3 (education and filter intervention), with 14 participants completing the posttest and two being lost to follow-up. All 15 participants assigned to group 4 (control group) completed the posttest.

The effects of the interventions on water testing were interesting. Throughout the study period, only one new water test was reported by participants. However, the test was not performed based on the intervention. The participant (in group 2 –filter only) found water spots on their car and decided to test the mineral concentration of their water.

Participants receiving the educational materials (groups 1 & 3) generally regarded the educational materials as helpful and thought-provoking at least in the short-term, though not enough to prompt a water test. One participant stated:

“*You talking to me, and the information was helpful. It brought stuff to my attention that I didn’t know or never thought about*.”

Another participant summed up the effect of the educational materials, stating:

“*Lots of information. It got me thinking, but then I forgot*.”

Overall, the intervention did not have an effect on water treatment. There was a positive association between membership in the filter-only group and water treatment. However, this was not significant at the p<0.05 level (p = 0.066; OR = 1.849; 95% CI = -0.125–3.823) (Table 5).

**Table 5:** Intervention effect on treatment.

Group	Odds Ratio	Std. Error	P Value	95% CI
1 Education	0.014	0.814	0.986	-1.582–1.610
2 Filtration	1.849	1.007	0.066	-0.125–3.823
3 Education and filtration	0.129	0.819	0.874	-1.475–1.735
4 (Reference Group)	-	-	-	-

Adjusted for missing data using multiple imputation method.

Models adjusted for clustering at the individual level.

None of the interventions had a significant effect on the constructs of risks or attitudes. The filter-only intervention had a significant effect on abilities (*b* = .831, 95% CI [-0.031, 1.630], p = 0.042) and self-regulation (*b* = 1.073, 95% CI [0.276, 1.870], p = 0.009) (Table 6). Likewise, the education plus filter intervention had a significant effect on abilities (*b* = .836, 95% CI [0.064, 1.609], p = 0.034) and self-regulation (*b* = .931, 95% CI [0.148, 1.714], p = 0.021). The education-only intervention did not significantly affect any of the assessed psychosocial factors.

**Table 6:** Intervention effect on psychosocial factors.

Construct	Group	Coefficient	Std. Error	P Value	95% CI
Risk	1	0.259	0.174	0.143	-0.090–0.608
Ref: Group 4					
	2	0.089	0.153	0.564	-0.218–0.396
	3	-0.033	0.133	0.801	-0.301–0.233
Attitudes	1	-0.042	0.152	0.782	-0.347–0.262
Ref: Group 4					
	2	-0.232	0.195	0.240	-0.623–0.159
	3	-0.100	0.164	0.544	-0.430–0.229
Norms	1	-0.149	0.239	0.536	-0.627–0.329
Ref: Group 4					
	2	0.191	0.296	0.521	-0.402–0.785
	3	-0.394	0.235	0.099	-0.865–0.076
Abilities	1	-0.175	0.348	0.617	-0.875–0.524
Ref: Group 4					
	2	0.831	0.397	**0.042**	0.031–1.630
	3	0.836	0.385	**0.034**	0.064–1.609
Self-regulation	1	0.276	0.352	0.437	-0.429–0.982
Ref: Group 4					
	2	1.073	0.397	**0.009**	0.276–1.870
	3	0.931	0.391	**0.021**	0.148–1.714

Note: Separate regressions were conducted for each assessed psychosocial construct. Adjusted for missing data using multiple imputation method.

Group1: Education intervention

Group 2: Filtration intervention

Group 3: Education and filtration intervention

The primary objective of this study was to evaluate, in practical terms, the effects of three interventions (education, the provision of water filters, and the combination of education and filters) on water testing and treatment relative to a control group. The educational materials proved to be ineffective in prompting participants to test their water, while an increase in intent to test was observed among participants receiving education only. No new tests were conducted during the three-month study period. The increase in intent could signal that, with more frequent cues to action, water testing could increase among well owners (Table 7).

**Table 7:** Effects of interventions on testing, treatment, and key indicators.

Group	Test Since Study Began	Sought Additional Information	% Increase Treatment	% Increase Commitment to Treatment
1	0	0	12%	+ 10%
2	1	0	+ 59%	+ 74%
3	0	0	+ 24%	+ 64%
4	0	0	+ 11%	- 5%

The results of this study found that providing free water filtration systems does not result in universal use. Of those receiving a filter, 40% did not use it. However, those that used the filters liked them and demonstrated commitment to continued use. The filter-only group saw a 59% increase in treatment use and a 74% increase in commitment to treatment. The education and filter group saw more modest gains in treatment use (24%) and commitment to treatment (64%). The discrepancy in filter use between the two groups can most likely be explained by the small sample size, though the overall percentage of those choosing not to use the filters is concerning (Table 7). The commitment to continue using the filters is encouraging, as a recent study found that while 76% of participants used filtration systems, less than half of participants (48%) reported liking the systems enough to continue using [36].

### Barriers to the use of household water treatment systems

Analysis of the open-ended questions revealed three themes when identifying barriers to the use of filtration systems: beliefs, knowledge, and functionality (Table 8).

**Table 8:** Themes and subthemes identified.

Theme	Subtheme	Mentions
Functionality	No Hot Water	1
	Incompatible	3
	Bulky	6
	Flow Rate	3
Beliefs	No Need/ Purity of Well Water	2
	Priority	4
Knowledge	Knowledge	3
Convenience	Easy to Install	8
	Easy to Use	8
Peace of Mind	Protection	12
	Idea of It	4

#### Beliefs

Beliefs, in this context, refer to the beliefs in the purity and natural high quality of groundwater. Within this theme, two subthemes emerged, the purity of well water and priority, both of which are interrelated. Two participants stated that due the lack of contaminants and high quality of their well water, they did not need a filter. One participant gave the filter to his son who moved to the city and is now on municipal water, stating:

“*I felt like him going to the city, he needed one. Water in the ground, less contamination, is better than the city. Could be wrong, I probably am, but that’s just what I’ve grown up knowing. Contamination, which is a big risk, but I don’t live by a nuclear power plant. I mean, it aint killed me yet. Yeah man, it’s just how I think. And I could be wrong*.”

Some participants did not explicitly mention the purity or quality of well water, though it did implicitly prevent the use of the filter. For these participants, they did not prioritize installing the system.

“*I’m not at home that much. I just never got around to installing it*.”

And

“*I didn’t even make a conscious decision. I’m busy as hell. I can’t. I don’t have time to deal with that right now. Honestly, it’s not at the top of my priority list. We have to set priorities and try to get it done. So right now, I had, I hadn’t had time to think about it*.”

One participant summed up the theme of beliefs as a barrier, stating:

“*I just hadn’t had time to put it on there or saw the need for it, so I didn’t get to put it on*.”

#### Knowledge

Like beliefs, knowledge was identified as a barrier to the use of filtration systems. In this context, knowledge refers to information on water quality/ environmental science, how treatment systems work and the protections they offer. Inadequate knowledge was present in the responses of some participants who stated their well water was safe due to the location of the well and the depth of their well. Furthermore, some participants did not understand what contaminants the filter removed.

“*Because I’ve already got a filter, uh, hooked up in my house. Well, actually it’s a sediment filter… just didn’t feel a need then to use it. “*

A participant (who initially used the filter and then purchased a sediment filter) summed up the theme of knowledge by stating:

“*Uh, it’s my impression that there are no contaminants in the water and the only thing it would be filtering out would be calcium. Cause of the water, if the water taste fine, I’d know. It’s deep water, the well, and water is deep… so it didn’t seem to be of any benefit*.”

#### Functionality

The final barrier identified was functionality, however, it was not found to prevent filter use among most participants. Elements of functionality (size, flow rate) were aspects of the systems that participants didn’t like. Size was the most mentioned dislike of the filtration systems, which could hinder sink operations:

“*It was just a little cumbersome and just not convenient. That’s the best way to put it. The bulkiness*.”

And

“*I guess it’s a little bulky for washing dishes*.”

Like size, the slow flow rate was mentioned by a few participants:

“*Well, the water comes out kind of slowly, but you get used to that*.”

And

“*I mean, it is slow, but I mean, that’s not an issue*.”

#### Facilitators of the use of household water treatment systems

Analysis of the open-ended questions revealed two themes when identifying facilitators of the use of water treatment systems: peace of mind and convenience (Table 8).

#### Peace of mind

Peace of mind refers to the feeling of comfort that comes from using the filtration system and has two subthemes: protection and the promise of added value.:

“*I really don’t care one way or the other, but we do it for the kids. You know, we protect the children*.”

And

“*I kind of, you know, feel safer*.”

And

“*I like that it’s cleaner, that it takes the bad stuff out. I would say that… to me, the big thing is the peace of mind knowing I’ve got that extra step in place to protect my family*.”

Other participants did not specifically state a reason(s) for using the system. They simply liked the idea of using, almost as a value add:

“*I guess you just feel like it’s a little cleaner, like you’re doing a little more…”*

and

“*The, well, just the idea more so than anything is that it’s filtering it before I drink it*.”

#### Convenience

Like peace of mind, convenience was identified as a facilitator of the use of treatment systems. Convenience refers to the ease of use and easy installation of the treatment systems. Participants commented that the systems were not complicated and were easy to get used to using:

“*Every time I get a glass of water, I looked at the little light on it to make sure it’s still good…it kind of folds out of the way*.”

And

“*It wasn’t anything complicated*.”

Finally, participants described the systems as being easy to install, promoting the use of the systems:

“*Install was very, it just snapped on there or screwed on there. It was very, very easy. I had it done in two minutes*.”

And

“*It’s easy to find their filters and install is easy*.*”*

And

“*… it’s the convenience of it, how it attaches with one snap… she’s very easy to put on and off, that was probably an advantage of that little thing. Plus, it’s really not that large and it really doesn’t, you know, keep you from doing things around the sink. You just kind of flip it to the back, to the side out of the way*.”

## Conclusion

The results of this study suggest that the current approach of local governments to educate well owners on stewardship behaviors may be ineffective. The messaging “protecting yourself and others” has merit, as seen in the facilitating subtheme of protection stated by participants using the water filter. However, the tangible benefit of water testing and subsequent benefit of treatment when needed, was not effectively communicated to the participants, nor were the possible repercussions of failing to test and treat well water.

New approaches and strategies to educating well owners on the need and benefits of stewardship behaviors are needed. The traditional message of protection speaks to some well owners but is not powerful enough to override the deep-seated beliefs around the purity of groundwater or the cultural and community norms that influence their decisions around stewardship behaviors. These two factors are extremely difficult to change and require immense community-engaged work from a programming perspective.

A possible new approach could be promoting these behaviors as an investment in their livelihoods and future. Relaying the economic advantages of protecting, maintaining, and monitoring their well and water may resonate with a greater percentage of the target population, as money affects everyone’s lives. Providing cost-benefit analyses on annual water testing and regular maintenance at the state or regional level (depending on data availability and known occurrences of contaminants) could provide well owners with accurate, operational intelligence needed to make an informed decision that is absent of any political or personal beliefs. Future research should include assessing the effectiveness of such improved programming at the local and national levels, targeting diverse communities, and culturally appropriate interventions that can elevate community buy-in when implementing such interventions.

This study has limitations, including the small sample size, and duration of the study. The small sample size may affect the true influence psychosocial factors have on testing and treatment behaviors. Likewise, the barriers and facilitators to HWTS use found in this study may not be associated with the long-term. The participants had knowledge of the future data collection points which could have influenced the use of the HWTSs.

Water quality data from the study area (collected by homeowners, researchers, local government, etc.) is limited. While some participants indicated having previously tested their water, no results were shared with the research team. Future studies on the local water quality will add context to the results and influence future testing and treatment interventions.

Finally, the results of this study may not be generalizable to the private well owner population in larger geographical locations. Attitudes and beliefs regarding well water and the environment may differ from those in other regions of the country. Likewise, environmental health literacy and awareness of water quality issues differ across regions.

## Supporting information

S1 File Pre test and post test questions. (PDF)

S2 File Department of public health’s well water Brochure. (PDF)

S3 File USEPA’s well water educational document. (PDF)
