# Supplementary material for: Private well water stewardship in rural Georgia
Source: PLoS One. 2024 Sep 19;19(9):e0307281. doi: 10.1371/journal.pone.0307281 (PMC11412682; doi:10.1371/journal.pone.0307281)
Supplement: S1 File — (PDF) [file pone.0307281.s001.pdf]

**Pretest**

Participant________________

**Well characteristics and location**

1. Type of Well
   Borehole_____ Tube_____ Other____________________

2. Year of construction__________

3. Depth of well________________

4. What type of pipes distribute the well water?
   _____Lead _____Mix
   _____PVC _____Do not know
   _____Steel

5. Was your well installed by a licensed contractor?

6. Is your well located 150 feet or less from a cesspool or a seepage pit?

7. Is your well located 100 feet or less from any of the following (check all that apply)?
   _____Septic tank absorption field _____Cemetery
   _____Lake, pond, or freshwater stream _____Fuel storage
   _____Marsh or swamp or any animal enclosure
   _____Fertilizer or chemical storage

8. Is your well located 50 feet or less from a septic tank?

9. Is your well located 10 feet or less than a sewer line?

10. Is this a flooding zone?

11. Do you use any of the following on your land?
    _____Fertilizer
    _____Herbicides
    _____Fungicides
    _____Insecticides

**Testing**

12. Has your well water ever been tested by a lab for contaminants? _____

13. If yes, what tests were conducted?
    ________________________________________________________________________________
    ________________________________________________________________________________
    ________________________________________________________________________________
    ________________________________________________________________________________

14. Did you specifically require for certain tests? If so which one?
____________________________________________________________________________
____________________________________________________________________________
____________________________________________________________________________
____________________________________________________________________________

15. Date of last well water test
_____Within 1 year
_____Last 1-2 years
_____Last 2-5 years
_____Last 5-10 years
_____More than 10 years ago
_____Never

16. How often do you test your well water?
_____Annually
_____Every 1-2 years
_____Every 2-5 years
_____Every 5-10 years
_____More than 10 years
_____Never

17. Which, if any, biologicals or chemicals were found (check all that apply)?
_____Arsenic
_____Lead
_____Iron
_____Bacteria
_____Viruses
_____Nitrates
_____Phosphates
_____Pesticides
_____Protozoa
_____Others

**Treatment**

18. Do you currently treat the well water before use? _____

19. If yes, do you treat at the source (well) or at the point of use (in home, under the sink or faucet)_____________________________________________

20. If yes, what method or methods do you use (check all that apply)
_____Chlorine in well
_____Chlorine in home
_____Carbon filtration
_____Reverse osmosis
_____Distillation
_____Absorbent media
_____Refrigerator filtration system
_____Boiling

**Maintenance**

21. Do you perform routine maintenance on your water system? _____

22. If yes, which activities do you perform (check all that apply)?
_____Pipe flushing
_____Part replacement and updating
_____Preventative maintenance

| | Strongly disagree | Somewhat disagree | Neither agree nor disagree | Somewhat agree | Strongly agree |
|---|---|---|---|---|---|
| **1. Well water quality can change over time** | 1 | 2 | 3 | 4 | 5 |
| **2. Wells in this area are at risk of contamination** | 1 | 2 | 3 | 4 | 5 |
| **3. We can be exposed to dangerous chemicals and microbes through water** | 1 | 2 | 3 | 4 | 5 |
| **4. I am at risk for drinking contaminated water** | 1 | 2 | 3 | 4 | 5 |
| **5. My untreated well water is safe to drink** | 1 | 2 | 3 | 4 | 5 |
| **6. I am not concerned with my well water because I have been drinking it for a long time with no health problems** | 1 | 2 | 3 | 4 | 5 |
| **7. Exposure to contaminated water results in considerable health risks** | 1 | 2 | 3 | 4 | 5 |
| **8.The risks of drinking untreated well water are overblown** | 1 | 2 | 3 | 4 | 5 |
| **9. Arsenic-related health effects are likely to be serious** | 1 | 2 | 3 | 4 | 5 |
| **10. Wells provide high quality water** | 1 | 2 | 3 | 4 | 5 |

| | Strongly disagree | Somewhat disagree | Neither agree nor disagree | Somewhat agree | Strongly agree |
|---|---|---|---|---|---|
| **11. Regular well water testing is affordable** | 1 | 2 | 3 | 4 | 5 |
| **12. Treating well water is expensive** | 1 | 2 | 3 | 4 | 5 |
| **13. Well water taste better than municipal/ city water** | 1 | 2 | 3 | 4 | 5 |
| **14. Municipal water has a bad smell** | 1 | 2 | 3 | 4 | 5 |
| **15. My friends regularly test their well water** | 1 | 2 | 3 | 4 | 5 |
| **16. My friends regularly treat their well water** | 1 | 2 | 3 | 4 | 5 |
| **17. My relatives regularly test their well water** | 1 | 2 | 3 | 4 | 5 |
| **18. My relatives regularly treat their well water** | 1 | 2 | 3 | 4 | 5 |
| **19. I believe most of my neighbors regularly test their well water** | 1 | 2 | 3 | 4 | 5 |
| **20. I believe my neighbors regularly treat their well water** | 1 | 2 | 3 | 4 | 5 |

| | Strongly disagree | Somewhat disagree | Neither agree nor disagree | Somewhat agree | Strongly agree |
|---|---|---|---|---|---|
| **21. My friends expect me to regularly test my well water** | 1 | 2 | 3 | 4 | 5 |
| **22. My friends expect me to treat my well water before drinking** | 1 | 2 | 3 | 4 | 5 |
| **23. My relatives expect me to regularly test my well water** | 1 | 2 | 3 | 4 | 5 |
| **24. My relatives expect me to treat my well water before drinking** | 1 | 2 | 3 | 4 | 5 |
| **25. My neighbors expect me to regularly test my well water** | 1 | 2 | 3 | 4 | 5 |
| **26. My neighbors expect me to treat my well water before drinking** | 1 | 2 | 3 | 4 | 5 |
| **27. I am personally responsible for the management of my well** | 1 | 2 | 3 | 4 | 5 |
| **28. I am personally responsible for ensuring the quality of my well water** | 1 | 2 | 3 | 4 | 5 |
| **29. I know who to contact to get my well water tested** | 1 | 2 | 3 | 4 | 5 |
| **30. I know what to test my well water for** | 1 | 2 | 3 | 4 | 5 |

| | Strongly disagree | Somewhat disagree | Neither agree nor disagree | Somewhat agree | Strongly agree |
|---|---|---|---|---|---|
| **31. I know how to treat my well water** | 1 | 2 | 3 | 4 | 5 |
| **32. Finding someone to test my well water is easy** | 1 | 2 | 3 | 4 | 5 |
| **33. I am confident I can treat my well water before drinking** | 1 | 2 | 3 | 4 | 5 |
| **34. I plan to have my well water tested in the next year** | 1 | 2 | 3 | 4 | 5 |
| **35.I plan on treating my well water before drinking** | 1 | 2 | 3 | 4 | 5 |
| **36. I want to test my well water, but keep forgetting to** | 1 | 2 | 3 | 4 | 5 |
| **37. I can remember to treat my well water before drinking it** | 1 | 2 | 3 | 4 | 5 |
| **38. I am committed to treating my well water before drinking it** | 1 | 2 | 3 | 4 | 5 |
| **39. I am committed to annually testing my well water** | 1 | 2 | 3 | 4 | 5 |

40. What year were you born? _______________

41. What is your gender? __________________

42. How would you classify yourself in terms of race and ethnicity? ________________

43. What is the highest level of formal education you have completed?

_____Some high school or less _____High school diploma or GED

_____Some college _____Technical or community college

_____Bachelor's degree _____Graduate degree

44. What is your employment status?

_____Work full-time _____Work part-time

_____Retired _____Unemployed

_____Homemaker

45. What was your total gross household income in 2019?

_____under $20,000 _____$20,000 - $35,000

_____$36,000 - $51,000 _____$52,000 - $67,000

_____$68,000 - $83,000 _____More than $100,000

_____$84,000 - $100,000

**Post Test Group 2 (Filtration Group)**

Participant ID #_______________

**Testing**

1. Have you tested your well water since the study began? _____

2. If yes, why did you decide to test your well water?
   ____________________________________________________________
   ____________________________________________________________
   ____________________________________________________________
   ____________________________________________________________

3. Did you request that specific contaminants be tested for and if so, which ones and why?
   ____________________________________________________________
   ____________________________________________________________
   ____________________________________________________________
   ___________________________________________

**Treatment**

4. Did you use the filtration system provided? _____

5. If no, why did you not use it?
   ____________________________________________________________
   ____________________________________________________________
   ____________________________________________________________
   ____________________________________________________________

6. How many times did you replace the filter? _____

7. Did you use the filtration system for the duration of the study? _____

8. If YES, what did you like about the filtration system?
   ____________________________________________________________
   ____________________________________________________________
   ____________________________________________________________
   ____________________________________________________________

9. If YES, what did you dislike about filtration system?
   ____________________________________________________________
   ____________________________________________________________
   ____________________________________________________________
   ____________________________________________________________
   ____________________________________________________________

10. If No, what did you dislike about filtration system?
________________________________________________________________________
________________________________________________________________________
________________________________________________________________________
________________________________________________________________________
________________________________________________________________________

11. If No, what did you like about filtration system?
________________________________________________________________________
________________________________________________________________________
________________________________________________________________________
________________________________________________________________________
________________________________________________________________________

12. If YES, do you intend on continuing to use the filtration system? _____

13. If NO, why did you not continue using the filtration system?
________________________________________________________________________
________________________________________________________________________
________________________________________________________________________
________________________________________________________________________

14. In addition to the filter provided, do you currently treat the well water before use? _____

15. If yes, what method or methods do you use (check all that apply)

_____Chlorine in well
_____Chlorine in home
_____Filtration (faucet or pitcher)
_____Reverse osmosis
_____Distillation
_____Absorbent media
_____Refrigerator filtration system
_____Boiling

16. Did you look for additional information after reading material? ______

**Maintenance**

17. Do you perform routine maintenance on your water system? _____

18. If yes, which activities do you perform (check all that apply)?

_____Pipe flushing
_____Part replacement and updating
_____Preventative maintenance

| | Strongly disagree | Somewhat disagree | Neither agree nor disagree | Somewhat agree | Strongly agree |
|---|---|---|---|---|---|
| **1. Well water quality can change over time** | 1 | 2 | 3 | 4 | 5 |
| **2. Wells in this area are at risk of contamination** | 1 | 2 | 3 | 4 | 5 |
| **3. We can be exposed to dangerous chemicals and microbes through water** | 1 | 2 | 3 | 4 | 5 |
| **4. I am at risk for drinking contaminated water** | 1 | 2 | 3 | 4 | 5 |
| **5. My untreated well water is safe to drink** | 1 | 2 | 3 | 4 | 5 |
| **6. I am not concerned with my well water because I have been drinking it for a long time with no health problems** | 1 | 2 | 3 | 4 | 5 |
| **7. Exposure to contaminated water results in considerable health risks** | 1 | 2 | 3 | 4 | 5 |
| **8.The risks of drinking untreated well water are overblown** | 1 | 2 | 3 | 4 | 5 |
| **9. Arsenic-related health effects are likely to be serious** | 1 | 2 | 3 | 4 | 5 |
| **10. Wells provide high quality water** | 1 | 2 | 3 | 4 | 5 |

| | Strongly disagree | Somewhat disagree | Neither agree nor disagree | Somewhat agree | Strongly agree |
|---|---|---|---|---|---|
| **11. Regular well water testing is affordable** | 1 | 2 | 3 | 4 | 5 |
| **12. Treating well water is expensive** | 1 | 2 | 3 | 4 | 5 |
| **13. Well water taste better than municipal/ city water** | 1 | 2 | 3 | 4 | 5 |
| **14. Municipal water has a bad smell** | 1 | 2 | 3 | 4 | 5 |
| **15. My friends regularly test their well water** | 1 | 2 | 3 | 4 | 5 |
| **16. My friends regularly treat their well water** | 1 | 2 | 3 | 4 | 5 |
| **17. My relatives regularly test their well water** | 1 | 2 | 3 | 4 | 5 |
| **18. My relatives regularly treat their well water** | 1 | 2 | 3 | 4 | 5 |
| **19. I believe most of my neighbors regularly test their well water** | 1 | 2 | 3 | 4 | 5 |
| **20. I believe my neighbors regularly treat their well water** | 1 | 2 | 3 | 4 | 5 |

| | Strongly disagree | Somewhat disagree | Neither agree nor disagree | Somewhat agree | Strongly agree |
|---|---|---|---|---|---|
| **21. My friends expect me to regularly test my well water** | 1 | 2 | 3 | 4 | 5 |
| **22. My friends expect me to treat my well water before drinking** | 1 | 2 | 3 | 4 | 5 |
| **23. My relatives expect me to regularly test my well water** | 1 | 2 | 3 | 4 | 5 |
| **24. My relatives expect me to treat my well water before drinking** | 1 | 2 | 3 | 4 | 5 |
| **25. My neighbors expect me to regularly test my well water** | 1 | 2 | 3 | 4 | 5 |
| **26. My neighbors expect me to treat my well water before drinking** | 1 | 2 | 3 | 4 | 5 |
| **27. I am personally responsible for the management of my well** | 1 | 2 | 3 | 4 | 5 |
| **28. I am personally responsible for ensuring the quality of my well water** | 1 | 2 | 3 | 4 | 5 |
| **29. I know who to contact to get my well water tested** | 1 | 2 | 3 | 4 | 5 |
| **30. I know what to test my well water for** | 1 | 2 | 3 | 4 | 5 |

| | Strongly disagree | Somewhat disagree | Neither agree nor disagree | Somewhat agree | Strongly agree |
|---|---|---|---|---|---|
| **31. I know how to treat my well water** | 1 | 2 | 3 | 4 | 5 |
| **32. Finding someone to test my well water is easy** | 1 | 2 | 3 | 4 | 5 |
| **33. I am confident I can treat my well water before drinking** | 1 | 2 | 3 | 4 | 5 |
| **34. I plan to have my well water tested in the next year** | 1 | 2 | 3 | 4 | 5 |
| **35.I plan on treating my well water before drinking** | 1 | 2 | 3 | 4 | 5 |
| **36. I want to test my well water, but keep forgetting to** | 1 | 2 | 3 | 4 | 5 |
| **37. I can remember to treat my well water before drinking it** | 1 | 2 | 3 | 4 | 5 |
| **38. I am committed to treating my well water before drinking it** | 1 | 2 | 3 | 4 | 5 |
| **39. I am committed to annually testing my well water** | 1 | 2 | 3 | 4 | 5 |
