# Supplementary material for: Private well water stewardship in rural Georgia
Source: PLoS One. 2024 Sep 19;19(9):e0307281. doi: 10.1371/journal.pone.0307281 (PMC11412682; doi:10.1371/journal.pone.0307281)
Supplement: S2 File — (PDF) [file pone.0307281.s002.pdf]

IS MY WELL WATER SAFE?

For homes that use private water wells, there can be certain risks if the well is not properly maintained and the water routinely tested. The water in your well can become contaminated from various chemical and biological hazards and make you sick.

Since there are no federal or state regulations for water testing or maintaining private wells, it is the well owner's responsibility to make sure their water is safe to drink. Well water may not be safe to drink if:

- you detect a difference in the taste, smell, or appearance of your well water
- your well casing or slab is damaged
- you have frequent and unexplained illnesses in your household
- you spill fertilizers, pesticides, oil, gasoline, or other toxic substances on the ground near the well or in the well
- your neighbors find toxic chemicals in their well water

Many contaminants do not affect the taste and appearance of your water, but will affect your health. Test your well water routinely to ensure the safety of your drinking water.

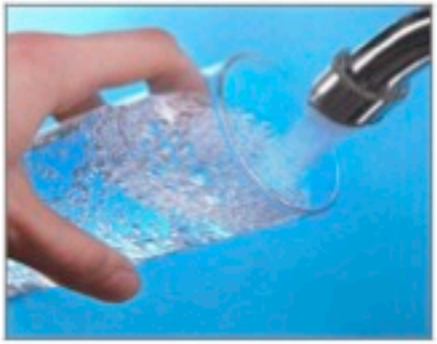

Typical Water Well Contamination Sources

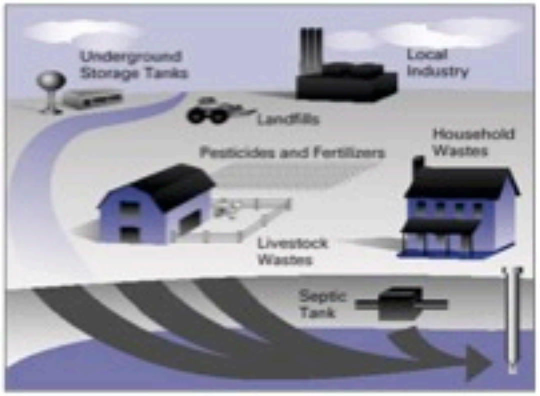

INSPECTION AND MAINTAINANCE

Inspect your well monthly for sources and entry points of contamination. Problems can exist with the land surface, the structure covering the well, the slab, the electrical conduit, the well cap, casing, and screen, and filtration equipment. Regular maintenance includes cleaning, repair, caulking holes and cracks, and keeping records.

Shallow and improperly constructed wells are especially vulnerable to contamination because of the potential for surface run-off and spills to get into your water supply. If you think your well might be contaminated or at risk, you should contact a professional to inspect your well.

Properly Abandon Wells

Unused water wells that are not properly abandoned leave dangerous open holes in the ground. Abandoned wells serve as direct channels for contamination to pass straight through to a drinking water source used by many people. To abandon a well, the owner must hire a licensed well driller.

POTENTIAL SOURCES OF WELL WATER CONTAMINATION

Pesticides, paints, cleaners, solvents, and many other toxic household chemicals must not be stored or applied near the well. Fuels like gasoline and oil are also poisons. Gasoline operated equipment such as lawn mowers can leak fuel onto the ground and travel to the water in your well. Also, any septic system and animal waste must be at least 100 feet from your well. These wastes can leach bacteria into the ground and contaminate your well water.

WELL WATER TESTING

Test all new wells. Test all wells for bacteria at least once per year (especially shallow wells), and for toxic chemicals every three years. To have your water tested, contact your county health department, county cooperative extension office or a certified private laboratory. If contaminants are found, a filtration or water treatment system can be installed.

University of Georgia
Cooperative Extension
Well Water Quality Information:
www.fcs.uga.edu/ext/housing

PROTECT YOUR HEALTH

- Perform well inspections monthly and maintenance when needed
- Keep toxic chemicals, septic systems, and animal wastes away from your well
- Test well water for bacteria and chemicals when it is installed and on a regular basis.
- If you have water quality concerns, install an appropriate home water treatment system
