# Supplementary material for: Private well water stewardship in rural Georgia
Source: PLoS One. 2024 Sep 19;19(9):e0307281. doi: 10.1371/journal.pone.0307281 (PMC11412682; doi:10.1371/journal.pone.0307281)
Supplement: S3 File — (PDF) [file pone.0307281.s003.pdf]

Protect Your
PRIVATE WELL

EPA

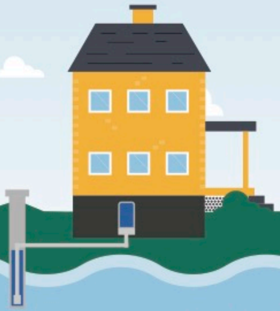

Many Americans receive their drinking water from a private well. These wells are not regulated by EPA or required to follow EPA's standards. If you use a private well, there are steps you can take to protect your water and ensure the safety of your drinking water.

How to PROTECT Your Well

TEST YOUR WELL WATER ANNUALLY

- Have your water tested annually for contaminants.
- Always use a state certified laboratory for testing.
- If contaminant levels exceed a drinking water standard, retest the water supply immediately and contact your public health department for assistance.

How to PREVENT Well Water Pollution

- Keep hazardous chemicals out of septic systems and away from your well
- Pump and inspect septic systems as recommended by your local health department
- Install a sanitary seal and slope the area around the well to drain surface runoff
- Hire a certified well driller for any new well construction or modification

How to REMEDIATE Your Well

If your well is contaminated or damaged, consult with your public health department, certified well inspector, and/or licensed well contractor to determine options for repair and remediation. Potential solutions include:

- Disinfectants to remove germs and microbes.
- Filters or other on-site treatment processes.
- Identifying a new water source.
- Digging a new, deeper well.

PAY ATTENTION to Your Well Surroundings and ASK QUESTIONS

- Find out about facilities that may pollute your drinking water.
- Attend planning or zoning meetings and ask questions about how your water sources will be protected.
- Ask to see a project's environmental impact statement. Check if water sources have been addressed.

HOW WELLS CAN BECOME CONTAMINATED

Wells can become contaminated by sources **around your well** and **your community**. A few examples include:

AROUND YOUR WELL

- Fuel tanks
- Lawns—fertilizers, pesticides, and herbicides
- Septic tanks and sewer lines
- Swimming pool chemicals

AROUND YOUR COMMUNITY

- Factories and industrial manufacturing
- Farms and animal feedlots
- New construction and mining operations
- Cemeteries

For more information, visit: **epa.gov/privatewells**
